# Role of the p38/AKT Pathway in the Promotion of Cell Proliferation by Serum Heat Inactivation

**DOI:** 10.3390/ijms242216538

**Published:** 2023-11-20

**Authors:** Huijun Geng, Rongnuo Li, Dingping Feng, Yuanyuan Zhu, Lu Deng

**Affiliations:** College of Animal Science and Technology, Northwest A&F University, Yangling, Xianyang 712100, China; 13183035829@163.com (H.G.); lrn000310@163.com (R.L.); fdpsci@163.com (D.F.); zyuanyuan929@163.com (Y.Z.)

**Keywords:** serum, heat inactivation, cell proliferation, p38/AKT pathway, ROS

## Abstract

Serum is a common biomaterial in cell culture that provides nutrients and essential growth factors for cell growth. Serum heat inactivation is a common treatment method whose main purpose is to remove complement factors and viruses. As serum contains many heat-labile factors, heat inactivation may affect cell proliferation, migration, differentiation, and other functions. However, the specific mechanism of its effect on cell function has not been studied. Thus, we investigate the exact effects of heat-inactivated FBS on the viability of various cells and explore the possible molecular mechanisms. We treated HCT116, HT-29, and HepG2 cell lines with heat-inactivated (56 °C for 30 min) medium, DMEM, or fetal bovine serum (FBS) for different times (0, 10, 15, 30, 60, or 90 min); we found that heat-inactivated FBS significantly promoted the viability of these cells, whereas DMEM did not have this effect. Moreover, heat-inactivated FBS stimulated cells to produce a small amount of ROS and activated intracellular signaling pathways, mainly the p38/AKT signaling pathway. These results indicate that heat-inactivated FBS may regulate the p38/AKT signaling pathway by promoting the production of appropriate amounts of ROS, thereby regulating cell viability.

## 1. Introduction

Serum is a commonly used material for cell cultures that contains the basic nutrients needed for cell growth and rich bioactive factors (such as growth factors, hormones, and peptides), which play an important role in regulating cell migration, proliferation, and differentiation [1,2]. In the early study of immune cell function, serum heat inactivation was used to inactivate complement factors and viruses in serum because activated complement factors can activate immune cells, such as lymphocytes and macrophages, and interfere with experimental studies [2]. Serum heat inactivation can also interfere with enzyme-linked immunosorbent assays and result in false-positive findings [3,4]. Previous studies have shown that serum heat inactivation can affect various biochemical indicators in serum, such as various metabolism-related enzymes [5,6,7]. In addition, because serum contains many heat-labile factors, it may have some effects on cell proliferation, migration, and differentiation [8,9]. However, the specific mechanism of its effect on cell function has not been studied. Therefore, in this study, we investigated the effect of serum heat inactivation on cell function and explored the mechanisms involved by investigating the effects on factors that are known to be involved in cell survival, proliferation, and function.

Previous studies have shown that p38, AKT, and mammalian target of rapamycin (mTOR) are important signaling pathways that regulate cell survival and proliferation [10,11,12]. mTOR is a protein kinase that acts as an ATP and amino acid sensor of nutrient levels and regulates cell growth [10]. mTOR is also regulated by AKT and p38 which, in turn, can regulate a range of kinases involved in cell growth, proliferation, and autophagy [13,14]. Activation of the mitogen-activated protein kinase (MAPK) signaling pathway is associated with a variety of cellular functions, including cell proliferation, differentiation, cell survival, and cell migration, through the activation of ERK, p38 MAPK, and JNK; in turn, they activate the transcription factors of kinases [15,16,17]. AKT, also known as protein kinase B, is a serine/threonine protein kinase. Originally discovered as a proto-oncogene, AKT plays a key role in regulating various cellular functions, including metabolism, growth, proliferation, survival, transcription, and protein synthesis [18]. AKT is one of the important substrates of mTORC2, and it promotes AKT activation by directly phosphorylating its hydrophobic motif Ser473, which is the site required for maximal AKT activation [19]. After AKT phosphorylation at Ser473, phosphatidylinositol-dependent protein kinase 1 (PDK1) further phosphorylated AKT at Thr308, resulting in full activation of AKT [3]. Sin1 is a component protein of mTORC2 that is essential for the assembly of mTORC2 and critical for the regulation of its substrate specificity [20]. Glycogen synthase kinase 3β (GSK3β) is a multifunctional serine/threonine kinase that regulates energy metabolism, cell growth, and apoptosis [21,22]. In addition, GSK3β is also a downstream substrate and effector of PI3K/AKT, and GSK3β is phosphorylated by PI3K/AKT to regulate the expression of downstream autophagy-related genes [23]. S6K is a component of the 40S ribosome. The mTORC1-S6K1 axis controls fundamental cellular processes, including transcription, translation, protein and lipid synthesis, cell growth/size, and cell metabolism. As a substrate of mTORC1, it is also affected by AKT and indirectly reflects the activity of AKT [24,25].

The p38 signaling pathway is commonly associated with oxidative stress, and reactive oxygen species (ROS) are an important source of oxidative stress [26]. ROS have long been considered as toxic molecules that lead to the accumulation of lipid peroxides and other cellular oxidative damage [27,28]. However, an increasing number of studies have shown that ROS act as secondary messengers for the maintenance of cell function and homeostasis and the regulation of phosphorylation, protein activation, and gene transcription [26,29]. In recent years, many studies have reported that physiological levels of ROS can activate specific biochemical reactions [30,31]. Low concentrations of ROS can promote the proliferation of some cells [31], and high concentrations of ROS can cause oxidative stress or even cell death [32]. Previous studies have found that hydrogen peroxide (H_2_O_2_) can activate many signaling pathways to promote the proliferation of normal cells and cancer cells [33]. Importantly, the ROS/p38/AKT signaling cascade was identified as an important signaling network for promoting proliferation [34]. Therefore, the mechanistic experiments in the present study focus on the role of this signaling cascade and their associated proteins in the effects of FBS heat inactivation on cell proliferation.

## 2. Results

### 2.1. Promotive Effect of Heat-Inactivated Medium on Cell Proliferation

**Figure 1:**
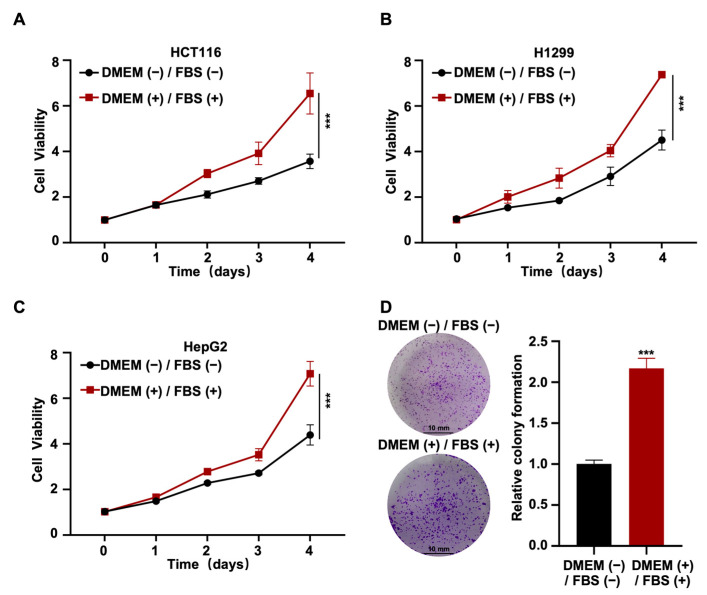
Promotive effect of heat-inactivated medium on cell proliferation. HCT116 (**A**), H1299 (**B**), and HepG2 (**C**) cells were treated with heat-inactivated medium for an indicated time; the viability of cells was detected by CCK8. (**D**) HCT116 cells were treated with heat-inactivated medium, and the viability of cells was detected by colony formation. Scale bar: 10 mm. All samples were normalized to cell number and conducted with three independent replicates. “+” represents “with heat-inactivated” and “−” represents “without heat-inactivated”. Data were analyzed using Student’s *t*-test and a two-way ANOVA test. *p*-values were considered statistically significant at *** *p* < 0.001.

To determine the effect of heat-inactivated medium (heat-inactivated DMEM + 10% heat-inactivated FBS) on cell function, we detected the cell viability of the HCT116 cell line cultured in heat-inactivated medium. The results of the cell viability assay experiments showed that the heat-inactivated medium was able to significantly affect the viability of cells. To prove the generalizability of this result, we repeated these viability experiments in human non-small cell lung cancer cells (H1299 cell line) and human hepatocarcinoma cells (HepG2 cell line) (Figure 1A–C) and obtained the same results. To confirm that heat-inactivated medium can affect cell viability, we performed a clone formation assay with the HCT116 cell line and obtained the same results (Figure 1D). Thus, based on these results, we were able to conclude that the heat-inactivated medium could promote cell proliferation.

### 2.2. Promotive Effect of Heat-Inactivated Medium on the AKT Pathway

**Figure 2:**
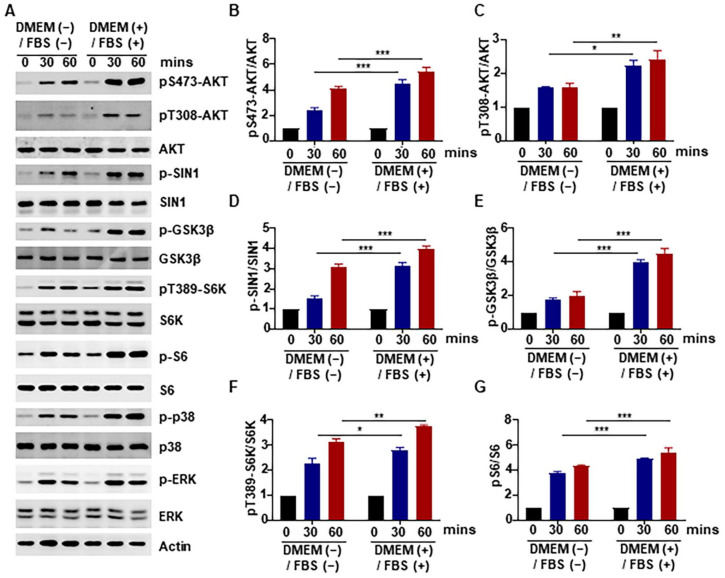
Promotive effect of heat-inactivated medium on the AKT pathway. (**A**–**G**). HCT116 cells were treated with heat-inactivated medium for 30 min and 60 min, respectively, and the levels of pS473-AKT, pT308-AKT, p-SIN1, p-GSK3β, pT389-S6K, p-S6, p-p38 and p-ERK, were detected by Western blotting. “+” represents “with heat-inactivated” and “−” represents “without heat-inactivated”. Data were analyzed by a two-way ANOVA test. * *p* < 0.05, ** *p* < 0.01, *** *p* < 0.001.

Heat-inactivated medium was found to significantly promote the phosphorylation of AKT on serine 473 and threonine 308. However, it was unclear whether this effect was limited to the phosphorylation and activation of AKT or whether it could affect downstream signaling pathways. Therefore, we examined the activity of proteins downstream of AKT, such as SIN1, GSK3β, and S6K [35,36,37] (Figure 2A–G), and found that the phosphorylation levels of these proteins were also significantly elevated. Therefore, these results reveal that the heat-inactivated medium not only significantly increased the phosphorylation of AKT but also promoted its downstream activity.

### 2.3. Promotive Effect of Heat-Inactivated FBS, but Not DMEM, on the AKT Pathway and Cell Viability

**Figure 3:**
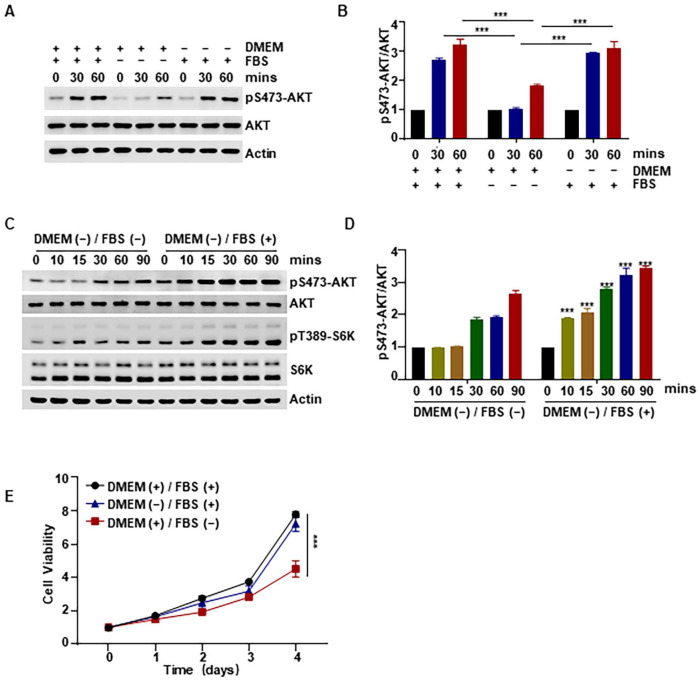
Promotive effect of heat-inactivated FBS, but not DMEM, on the AKT pathway and cell viability (**A**,**B**). HCT116 cells were treated with heat-inactivated medium, DMEM, or FBS for 30 min and 60 min, respectively, and the level of pS473-AKT was detected by Western blotting. (**C**,**D**) HCT116 cells were treated with heat-inactivated FBS for an indicated time, and the levels of pS473-AKT and pT389-S6K were detected by Western blotting. (**E**) HCT116 cells were treated by heat-inactivated medium, FBS, or DMEM for an indicated time; the viability of cells was detected by CCK8. “+” represents “with heat-inactivated” and “−” represents “without heat-inactivated”. Data were analyzed by a two-way ANOVA test. *** *p* < 0.001.

Next, in order to confirm that the observed effects on cell proliferation and AKT activity were attributable to the FBS and not to DMEM, we treated HCT116 cell lines with heat-inactivated medium, DMEM, or FBS. Heat-inactivated FBS (non-heat-inactivated DMEM + 10% heat-inactivated FBS) was found to activate AKT phosphorylation and showed a similar ability to the heat-inactivated medium; however, treatment with DMEM alone did not have this effect (Figure 3A,B). To further confirm the role of FBS, we used heat-inactivated FBS to treat HCT116 cells and found a significant increase in AKT and S6K phosphorylation levels (Figure 3C,D). At the same time, we examined the effect of heat-inactivated DMEM (heat-inactivated DMEM + 10% non-heat-inactivated FBS) or FBS on cell viability and found that it was indeed heat-inactivated FBS that promoted cell viability (Figure 3E). These findings confirm that heat-inactivated FBS promotes the AKT pathway and cell viability and that DMEM is not involved in these effects.

### 2.4. Role of p38 in the Effect of Heat-Inactivated FBS on the AKT Pathway and Cell Viability

**Figure 4:**
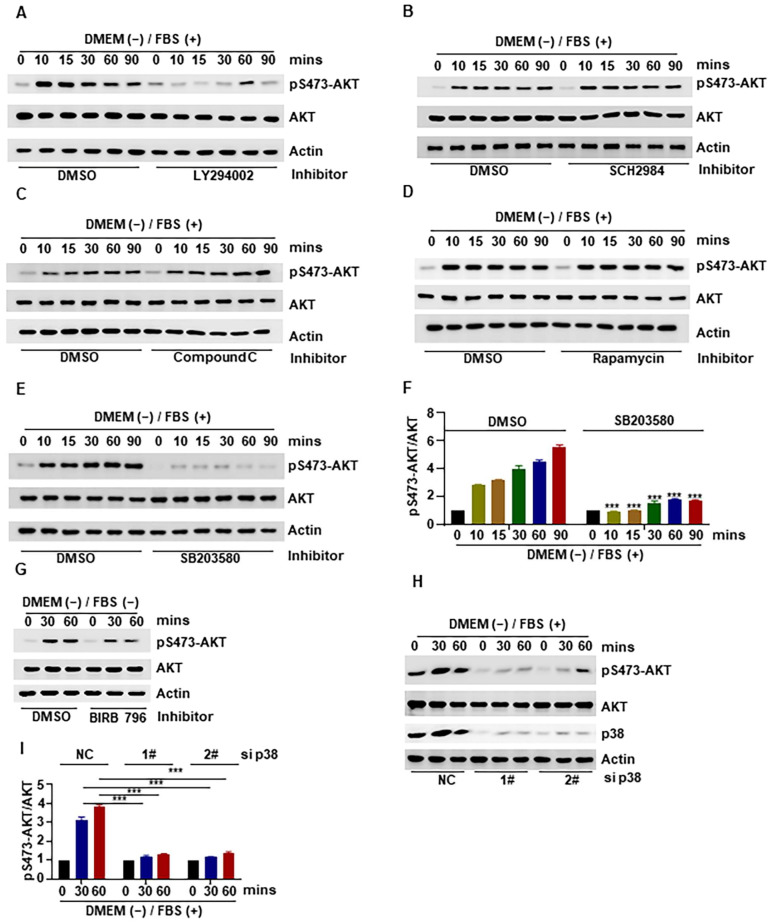
Role of p38 in the effect of heat-inactivated FBS on the AKT pathway and cell viability. (**A**–**G**). HCT116 cells were treated with heat-inactivated FBS with LY294002 (20 μM), SCH772984 (5 μM), compound C (4 μM), rapamycin (100 nM), SB203580 (10 μM), and BIRB 796 (10 μM) for an indicated time, and the level of pS473-AKT was detected by Western blotting. (**H**,**I**). After knockdown of p38 in HCT116 cells, the level of pS473-AKT was detected by Western blotting. “+” represents “with heat-inactivated” and “−” represents “without heat-inactivated”. Data were analyzed by a two-way ANOVA test. *** *p* < 0.001.

In the previous experiments (Figure 2), we found that heat-inactivated medium could activate the MAPK pathway and PI3K/AKT pathway. Therefore, we examined whether these effects were present when inhibitors against the following proteins were used: LY294002 (AKT inhibitor), SCH772984 (ERK inhibitor), compound C (AMPK inhibitor), rapamycin (mTORC1 inhibitor), and SB203580 (p38 MAPK inhibitor) (Figure 4A–E). The results showed that AKT phosphorylation was significantly inhibited in the presence of the AKT inhibitor LY294002 (Figure 4A) and the p38 inhibitor SB203580 (Figure 4E,F). This means that p38 might be involved in the effect of heat-activated FBS on cell viability and AKT. To further confirm the role of p38, we used the more specific inhibitor of p38 MAPK named BIRB 796, and the results showed that the phosphorylation of AKT at Ser-473 was inhibited by BIRB 796 (Figure 4G). To further demonstrate the effect of heat-inactivated FBS on the p38/AKT pathway, we also found that p38 knockdown significantly inhibited heat-inactivated FBS-mediated AKT activation by using siRNAs, which fully demonstrated the effect of heat-inactivated FBS on the p38/AKT pathway (Figure 4H,I). Based on these findings, we concluded that heat-inactivated FBS may promote the AKT pathway and cell viability through p38.

### 2.5. Role of ROS in the Effect of Heat-Inactivated FBS on AKT Signaling and Cell Viability

**Figure 5:**
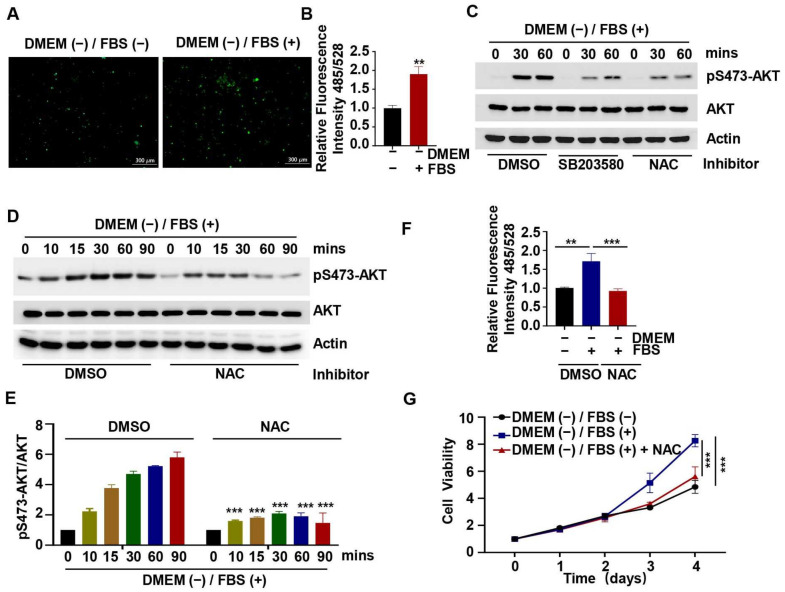
Role of ROS in the effect of heat-inactivated FBS on AKT signaling and cell viability. (**A**,**B**,**F**). HCT116 cells were treated with heat-inactivated FBS or NAC for 1 h, and ROS levels were measured. (**C**–**E**,**G**). HCT116 cells were treated with heat-inactivated FBS adding SB203580 or NAC (2 mM) for an indicated time; then, the level of pS473-AKT was detected by Western blotting, and the viability of cells was detected by CCK8. “+” represents “with heat-inactivated” and “− represents “without heat-inactivated”. Data were analyzed by a Student’s *t*-test and two-way ANOVA test. ** *p* < 0.01, *** *p* < 0.001.

Heat-inactivated FBS was found to promote ROS production in HCT116 cells (Figure 5A,B). To verify this result, we added SB203580 or NAC (an ROS scavenger) to the heat-inactivated FBS and found that SB203580 and NAC both significantly inhibited the phosphorylation of AKT at Ser473 (Figure 5C). To further confirm the role of NAC, HCT116 cells were treated with NAC for an indicated time (0, 10, 15, 30, 60, 90 min). As expected, the phosphorylation of AKT at Ser473 was significantly reduced (Figure 5D,E). Meanwhile, we found that the ROS levels were elevated with heat-inactivated FBS in HCT116 cells and significantly reduced by the addition of NAC (Figure 5F). In addition, we measured cell viability after the addition of NAC and found that NAC inhibited the pro-proliferation effect of heat-inactivated FBS (Figure 5G). Therefore, these findings imply that heat-inactivated FBS regulates ROS production to promote AKT signaling and cell viability.

## 3. Discussion

In the present study, we have examined the effects of heat-inactivated FBS on the growth of cells and examined the underlying mechanisms. These findings are important, as the underlying mechanisms have not been clearly elucidated yet.

Actually, our current work is trying to address the currently controversial point of some research using heat-inactivated FBS to culture cells but others using non-heat-inactivated FBS [38]. In fact, the main reasons why researchers use heat-inactivated FBS are as follows: (1) To remove mycoplasma contamination. However, the current development of technology has been able to completely remove mycoplasma from FBS [39] and, to some extent, heat inactivation can be dispensed with; (2) To eliminate the role of complement, which is largely due to the fact that the complement system affects the cultivation of immune cells. Nevertheless, it may be shown that it is not so necessary for non-immune cells. Moreover, FBS contains very little complement. Triglia and Linscott found that C1 and C6 in FBS were only 1–3% of adult animal serum. Other complement components were only 5–50% of adult animal serum, and C3, the major component of complement, was almost undetectable in FBS [40]; (3) Another factor that may be the main reason why many studies today do not use heat-inactivated FBS to culture cells is that the heat inactivation of FBS may destroy some heat-labile cytokines or components, which may be fatal for cell cultures. For these reasons, heat inactivation and non-heat inactivation are controversial in the field today, and the main issue we want to address in this work is the effect of heat inactivation and non-heat inactivation on the growth of cancer cells (non-immune cells); moreover, we evaluated the growth of cells under two scenarios (heat inactivation and non-heat inactivation). Our study found that heat-inactivated FBS could activate the p38/AKT pathway, which could promote cell growth to a certain extent. Therefore, we believe that heat inactivation of FBS is necessary, and this finding provides theoretical guidance for the selection of cell culture conditions.

Firstly, in our study, we selected human colorectal carcinoma cells (HCT116) from colon tissue, human non-small cell lung cancer cells (H1299) from lung tissue, and human hepatocarcinoma cells (HepG2) from liver tissue. Our data show that these cell lines (from different tissues or tumors) show a consistent response to heat inactivation of the culture medium (DMEM and FBS), which also confirms the generalizability of our conclusions (Figure 1). In agreement with our findings, in 1971, Balk et al. [41] observed that normal chick embryo fibroblasts differentiated and proliferated rapidly in serum-containing medium treated with heat (56 °C for 30 min), and the authors suspected that the effect was a result of changes in a platelet-releasing factor. Along similar lines, in 2004, Bruinink [1] found that heat inactivation of either calf serum or human serum promoted the proliferation of trabecular bone-derived progenitor cells. Furthermore, another study showed that the heat inactivation of calf serum enhanced cell attachment under fluid shear stress [42]. However, it has also been reported that heat-inactivated serum may not promote cell proliferation and migration. For example, Leshem et al. [43] demonstrated that heat-inactivated serum did not affect murine lymphocyte proliferation. Further, Van et al. [44] found that the ability of heat-inactivated serum to promote cell proliferation was not as strong as that of fresh serum that is not inactivated; these findings indicate the presence of heat-labile cell growth factors in human serum that may not be present in bovine serum. These studies seem to indicate that the effect of heat-inactivated serum on cell proliferation may differ between species. Thus, the decision to perform heat inactivation is very important when selecting serums for cell cultures, and our research provides a reference for this purpose.

In order to identify the molecular mechanisms underlying the observed effect of heat inactivation on cell viability in this study, we examined changes in the proteins associated with the cell proliferation-related AKT signaling pathway and found that there was an increase in the phosphorylation of AKT and several of its downstream proteins (Figure 2). This implies that the effect of medium heat inactivation on cell viability was brought about via increased phosphorylation and activation of AKT. Furthermore, we confirmed that these effects on AKT and cell viability were attributable to FBS and not to DMEM (Figure 3A,B). We also conducted mechanistic experiments with inhibitors of p38 and other proteins that might be involved, and the results showed that AKT activation was significantly inhibited by inhibition of p38 phosphorylation (Figure 4). In line with these findings, our previous study showed that p38 could regulate the activity of mTORC2 and the phosphorylation of AKT by phosphorylating SIN1 [45]; however, the exact mechanism needs further investigation. Nonetheless, the results demonstrate that heat-inactivated FBS exerts its growth-promoting effect through the p38/AKT pathway. Because the p38/MAPK signaling pathway is an important pathway in the recognition of oxidative stress [12], we examined the intracellular ROS content and found that heat-inactivated FBS could promote the production of a small amount of ROS (Figure 5A,B). Therefore, we hypothesized that heat-inactivated FBS induced ROS production. To test this hypothesis, we added NAC to heat-inactivated FBS and found that phosphorylation of AKT was inhibited in the presence of NAC. Therefore, we conclude that heat-inactivated FBS induces ROS production to promote the AKT signaling pathway and cell viability. Moreover, NAC was also found to restore the pro-proliferative effect of heat inactivation. In contrast, in the study by Van et al. [44], the addition of GSH did not restore the proliferation of human clonal T cells cultured in heat-inactivated serum. This is probably because GSH cannot easily cross the cell membrane, making its precursor substance NAC more stable and more effective at scavenging ROS than GSH [46].

To examine the mechanism further, we performed metabolomic sequencing and screened several specific metabolites from the metabolomic data. Moreover, we validated the effect of the metabolites 1-hydroxypyrene and dl-glutamine on the phosphorylation level of AKT. Unfortunately, we did not identify the key metabolites that could generate ROS and activate the p38/AKT pathway. In the future, we will continue to explore the effects of heat-inactivated FBS on the function of other cells and more detailed molecular mechanisms so as to provide guidance and suggestions for serum heat inactivation.

## 4. Materials and Methods

### 4.1. Reagents

Cell Counting Kit-8 (CCK8) (C0038) was purchased from Beyotime (Shanghai, China). LY294002 (S1105) and BIRB 796 (S1574) were obtained from Selleck Chemicals (Shanghai, China). *N*-acetylcysteine (NAC, A7250), rapamycin (V900930), SCH772984 (942183-80-4), SB203580 (HY-10256), and secondary antibodies were obtained from Sigma-Aldrich (St. Louis, MO, USA). Compound C (S7840) was obtained from Selleck Chemicals (Houston, TX, USA). Phosphate-buffered saline (PBS) and trypsin were purchased from HyClone (Logan, UT, USA). RPMI 1640, Dulbecco’s modified Eagle’s medium (DMEM), β-mercaptoethanol, penicillin, fetal bovine serum (FBS), and streptomycin were bought from Gibco (Grand Island, NY, USA). Primary antibodies against the following proteins were obtained from Cell Signaling Technology (Beverly, MA, USA): pT389-S6K (9234S/L), S6K (9202S), p-S6 (4858S), S6 (2217S), pS473-AKT (9271), pT308-AKT (4056), AKT (9272), p-SIN1 (114716), SIN1 (12860), p-p38 (4631), p38 (8690), p-ERK1/2 (4370), and ERK1/2 (4695). In addition, primary antibodies against pS9-GSK3β (ab75814) and GSK3β (ab32391) were obtained from Abcam (Cambridge, UK). β-actin was used as an internal control, and primary antibodies against actin were obtained from Proteintech (20536-1-AP, Wuhan, China).

### 4.2. Cell Culture

Human colorectal carcinoma cells (HCT116), human non-small cell lung cancer cells (H1299), and human hepatocarcinoma cells (HepG2) were purchased from National Science & Technology Infrastructure (NSTI; Shanghai, China). HCT116 and HepG2 cells were cultured in DMEM medium containing 10% FBS, and H1299 cells were cultured in RPMI 1640 medium containing 10% FBS in accordance with the ATCC guidelines. For heat inactivation, FBS was heated at 56 °C for 30 min. The heat-inactivated medium was “heat-inactivated DMEM + 10% heat-inactivated FBS”, heat-inactivated DMEM was “heat-inactivated DMEM + 10% non-heat-inactivated FBS”, and heat-inactivated FBS was “non-heat-inactivated DMEM + 10% heat-inactivated FBS”. Heat inactivation was performed at 56 °C for 30 min.

### 4.3. Cell Viability Assay

Cells were seeded at a density of 10^3^ per well in 96-well plates. After cell adhesion, the HCT116, H1299, and HepG2 cells were cultured in heat-inactivated medium. On days 0, 1, 2, 3, and 4, the original medium was replaced with fresh medium (100 μL) containing 10% CCK8 reagent and incubated for 2 h at 37 °C. Finally, a Synergy HT microplate reader (Bio-Tek, Winooski, VT, USA) was used to determine the absorbance of each well of the plate at 450 nm.

### 4.4. Colony Formation Assay

The medium containing 10% heat-inactivated FBS was added to 6-well plates containing 1000 to 2000 HCT116 cells and incubated for 7 days. The cells were washed three times with PBS, fixed with 4% paraformaldehyde solution at room temperature for 20 min, and stained with crystal violet solution at room temperature for 30 min. After staining, the cells were washed several times with PBS for 5 min each time at room temperature until the cell background was transparent; then, they were photographed and preserved.

### 4.5. siRNA Knockdown

Lipofectamine 2000 reagent (Invitrogen, CA, USA) and siRNA oligonucleotides were used to transfect HCT116 cells. The following siRNAs were used:

siRNA p38-1#: 5′-ATTTCTGTAGGAAATCACACG-3′

siRNA p38-2#: 5′-ACTTTGCATTGAACATATTCC-3′

### 4.6. Western Blot Assay

Western blotting analyses were performed as previously described [47]. Briefly, cells were harvested and suspended in radioimmunoprecipitation assay (RIPA) buffer on ice for 30 min after being washed with PBS. Proteins were separated by sodium dodecyl sulfate-polyacrylamide gel electrophoresis and transferred onto nitrocellulose membranes (0.45 μM, GE). Then, the membranes were incubated with primary antibody overnight and washed with PBS three times. Following this, the membranes were incubated with horseradish peroxidase-conjugated secondary antibody at room temperature for 2 h and washed with PBS–0.1% Tween-20 (PBST) three times. Finally, the proteins were detected using the Bio-Rad imaging system (Bio-Rad, Hercules, CA, USA), and ImageJ software (version 1.48, Wayne Rasband, Maryland, USA) was used to quantify protein abundance.

### 4.7. Detection of ROS

HCT116 cells were seeded in 96-well plates containing medium treated at 56 °C for 1 h. The plates were then loaded with dichlorodihydrofluorescein diacetate (R252; DOJINDO, Kumamoto, Japan) and incubated at 37 °C for 30 min. The plates were washed to remove excess fluorescence solution, and fluorescence intensity was detected with the Hybrid Multi-Mode Reader (Bio-Tek, Synergy H1, Winooski, VT, USA).

### 4.8. Statistical Analysis

Data analysis was performed in GraphPad Prism 9.0 software. All experiments were repeated at least three times, and the results were expressed as the mean ± SEM. For the unpaired one-tailed or two-tailed Student *t*-tests and one-way or two-way analysis of variances, *p*-values of less than 0.05 were considered to indicate statistical significance.

## 5. Conclusions

The current findings indicate that heat-inactivated FBS can promote the production of appropriate amounts of ROS, thereby regulating the p38/AKT signaling pathway through ROS and ultimately regulating cell viability. Further investigations are required to confirm and elucidate the details of this mechanism, especially with regard to which metabolites might be involved. These findings suggest that the process of FBS heat inactivation is very important for cell cultures, and the appropriate serum should be selected according to the characteristics of different cells and whether heat inactivation should be carried out or not.

## Data Availability

The data generated and analyzed in this study can be provided by the corresponding author upon reasonable request.
